# Novel heterozygous *VPS13A* pathogenic variants in chorea-neuroacanthocytosis: a case report

**DOI:** 10.1186/s12883-023-03398-x

**Published:** 2023-10-04

**Authors:** Xi Chen, Piao Zhang, Lijuan Wang, Yuhu Zhang

**Affiliations:** 1 Department of Neurology, Guangdong Neuroscience Institute, Guangdong Provincial People’s Hospital, Guangdong Academy of Medical Sciences, Southern Medical University, No. 106 Zhongshan Er Road, Guangzhou, 510080 China; 2 Shantou University Medical College, Shantou, China; 3 Guangzhou Key Laboratory of Diagnosis and Treatment for Neurodegenerative Diseases, Guangdong Provincial People’s Hospital (Guangdong Academy of Medical Sciences), Guangzhou, 510080 China

**Keywords:** Neuroacanthocytosis, Chorea, *VPS13A*, Movement disorders

## Abstract

**Background:**

Chorea-acanthocytosis (ChAc) is a rare hereditary autosomal recessive neurodegenerative disorder caused by pathogenic variants of the Vacuolar Protein Sorting 13 homolog A (*VPS13A*) gene. The variant spectrum of *VPS13A* has not been completely elucidated. This study reports two novel heterozygous *VPS13A* pathogenic variants in ChAc that expand the variant spectrum of *VPS13A*.

**Case presentation:**

We described a case of a 29-year-old man with typical clinical manifestations of ChAc, including chorea, orofacial lingual dyskinesia, vocal tics, elevated serum biochemical indicators, increased acanthocytes in peripheral blood, and caudate nucleus atrophy. Next-generation sequencing revealed two heterozygous variants of *VPS13A*: a nonsense variant (NM_033305.2: c.8215G > T, p. Glu2739Ter) and a deletion variant in the exons 25–31.

**Conclusion:**

The identified nonsense variant gives rise to premature translation termination, while the deletion variant is expected to cause a significant in-frame deletion of amino acid residues in the encoded protein. Both variants are considered to be pathogenic and result in loss-of-function proteins. These findings have implications for the genetic counseling of patients with *VPS13A* variants.

## Background

Chorea-acanthocytosis (ChAc) is a rare neurodegenerative disorder that is caused by pathogenic variants of the Vacuolar Protein Sorting 13 homolog A (*VPS13A*) gene, which is located on chromosome 9q21 and follows an autosomal recessive inheritance pattern [1]. ChAc presents with diverse clinical manifestations that typically occur in young adulthood, including involuntary movements such as generalized chorea, orofacial lingual dyskinesia, and vocal tics, as well as cognitive-psychiatric disorders, seizures, and peripheral neuropathy. Elevated serum biochemical indicators may be revealed by laboratory tests while an increased count of acanthocytes may be observed in a peripheral blood smear. Neuroimaging studies show atrophy of the caudate nucleus and reduced glucose utilization in the striatum, supporting the striatum as an area of primary dysfunction [2, 3]. The diagnosis of ChAc is based on typical clinical manifestations, laboratory findings, and neuroimaging with the exclusion of other types of acanthocytosis, such as abetalipoproteinemia (ABL), McLeod syndrome (MLS), Huntington’s disease-like 2 (HDL2), and pantothenate kinase-associated neurodegeneration (PKAN). Detection of biallelic variants in *VPS13A* will confirm the diagnosis.

The *VPS13A* gene encodes a large molecular protein called chorein, which is involved in the intracellular transport of transmembrane proteins and the sorting of vacuolar proteins [4]. There are fewer than 1000 cases that have been reported worldwide to date [5]. Although various pathogenic variants have been identified in previous cases, the variant spectrum of *VPS13A* is not fully understood. Herein, we discussed a case of a 29-year-old male who had been suffering from chorea and orolingual dyskinesia for 16 months. A genetic study using next-generation sequencing identified a combination of a heterozygous nonsense variant (NM_033305.2: c.8215G > T, p. Glu2739Ter) and a heterozygous deletion variant in the exons 25–31 of the *VPS13A* gene on chromosome 9q21 in the affected individual. Both variants were newly classified as pathogenic according to a literature review.

## Case presentation

A 29-year-old male, who had been suffering from repeated involuntary movements of the lips, tongue, and limbs for 16 months with aggravation for more than 4 months, was admitted to our hospital. The perioral involuntary movements included grimacing, grinning, chewing, sounding, and uncontrolled frequent tongue and lip biting. The appendicular involuntary movements included arms swinging, shrugging, stomping, and abnormal gait. These symptoms worsened over time and he developed an obvious gait disorder and frequent falls when visiting the clinic. The patient had a history of penicillin allergy but no history of drug exposure that would cause extrapyramidal dysfunction. The patient denied being born to consanguineous parents and denied similar cases in the family.

## Neurological physical examinations

Neurological physical examinations showed generalized chorea, dystonia in the extremities, orolingual dyskinesia with slurred speech, vocal tics and self-mutilation, and a shuffling gait. No exceptions were detected in the examinations of muscle volume, muscle strength, tendon reflex, sensation, and coordination. No Kayser-Fleischer (K-F) ring was detected in both corneas. Nearly no impairment in high cognitive function with a Mini-Mental State Examination (MMSE) scored 27/30 (junior college).

### Laboratory tests

**Fig. 1:**
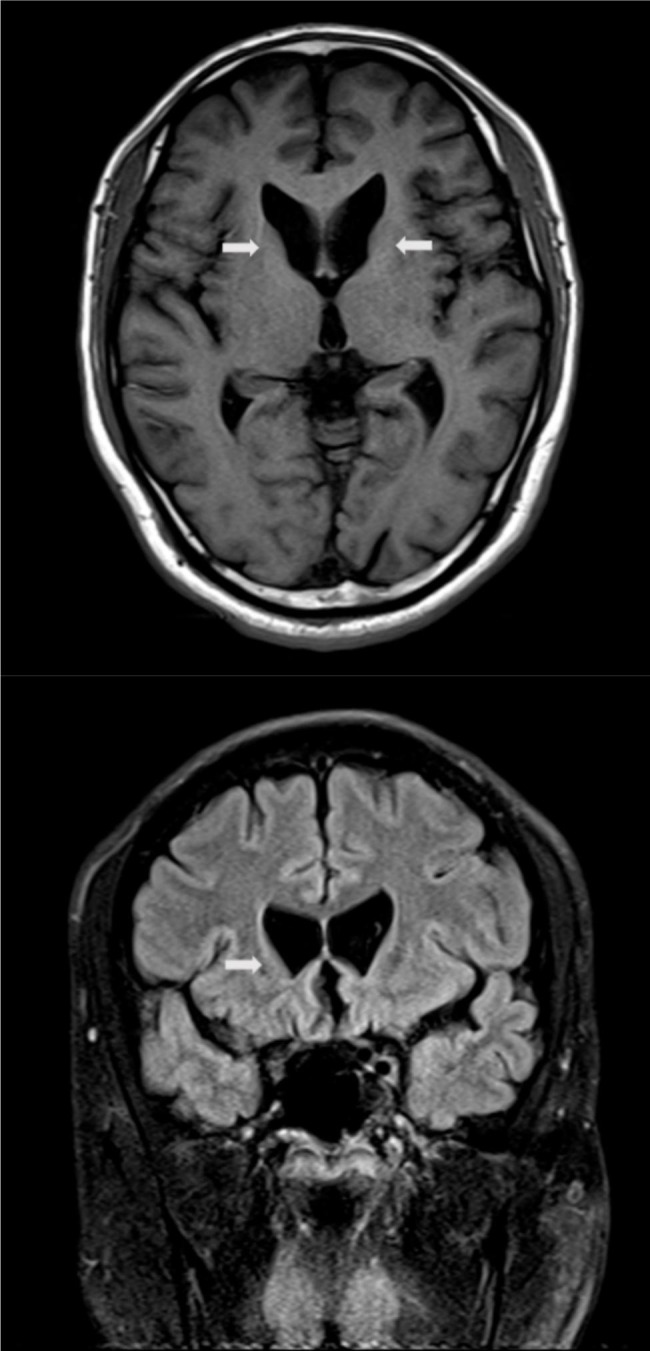
Magnetic resonance images show bilateral atrophy of the head of the caudate nucleus (arrows). (**A**: transverse, **B**: coronary)

Laboratory tests showed abnormal blood cell counts, elevated liver enzyme levels, and elevated creatine kinase levels. The results were as follows: 7.53 *10^12/L red blood cells ((4.30–5.80) *10^12/L)), 68.0fL mean red blood cell volume (82.0-100.0fL), 20.8pg mean red blood cell Hb content (27.0-34.0pg), 307 g/L mean red blood cell Hb concentration (316-354 g/L), 72U/L alanine aminotransferase (normal: 9-50U/L), 80U/L aspartate aminotransferase (normal: 15-40U/L), 2715.9 U/L creatine kinase (normal: 50-130U/L), 358.9 U/L lactate dehydrogenase (normal: 109-245U/L). No other significant exceptions were detected in the tests of blood, urine, stool, serum lipids, renal function, erythrocyte sedimentation rate, serum B12 levels, folic acid levels, serum copper, and ceruloplasmin. Electrocardiogram, echocardiogram, abdominal ultrasonogram, and electroencephalogram were normal. Acanthocytes were noted on a peripheral blood smear with a proportion ranged 6%. Broken red blood cells were also shown in the peripheral blood smear. Brain magnetic resonance imaging (MRI) showed atrophy of bilateral caudate nucleus heads (Fig. 1). Electromyography showed peripheral neurogenic lesions of both upper limbs, mainly involving the distal sensory axonal fibers.

### Genetic analyses and results

Genomic DNA was extracted from the patient’s peripheral blood and was subjected to both Sanger sequencing and Illumina NovaSeq high-throughput sequencing. Target sequencing panels were designed to include the following genes: *NKX2-1, SETX, AFG3L2, APP, APTX, AR, ATP1A2, ATXN10, BEAN1, CACNA1A, CACNB4, FGF14, HTT, ITPR1, JPH3, KCNA1, KCNC3, KCND3, PDYN, PRKCG, PRNP, PSEN1, PSEN2, SCN1A, SLC1A3, SPTBN2, TBP, TGM6, TTBK2, TTPA, XK, VPS13A, ADCY5, ATG7, HSD17B10, MED17, OPA3, PDE10A, PRRT2, TARDBP*, and *MTTP*. The sequencing services were provided by the KingMed Diagnostics Institute (Guangzhou, China), achieving at least 99% coverage of the target regions and a sequencing depth of 90×. The interpretation of variants was classified into 5 categories: pathogenic, likely pathogenic, uncertain significance, likely benign, and benign, according to the American College of Medical Genetics and Genomics guidelines (ACMG) [6].

**Fig. 2:**
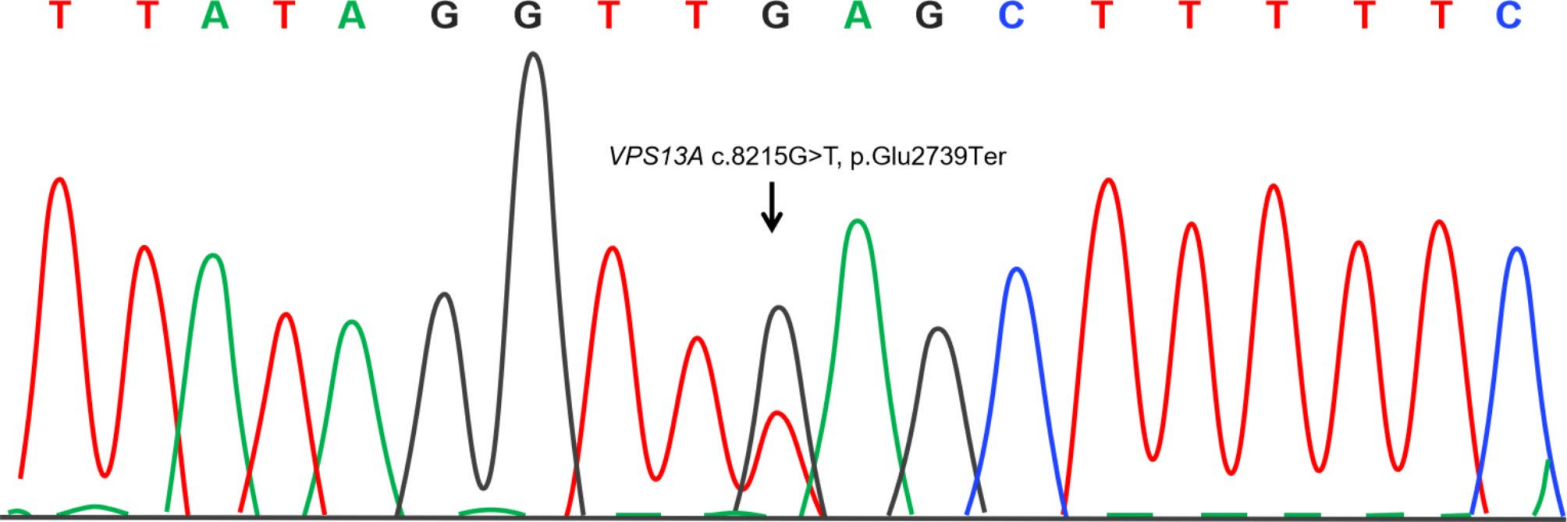
Sanger sequencing found a heterozygous variant (NM_033305.2: c.8215G > T, p. Glu2739Ter) of *VPS13A*

In the proband, two variants were identified in the *VPS13A* gene (reference sequence: NM_033305.2). First, a nonsense variant c.8215G > T (p. Glu2739Ter) (Fig. 2), which was predicted to change the amino acid at position 2739 of the encoded protein from glutamate to a stop codon, has not been reported in the database of HGMD, ESP6500, dbSNP or 1000genomics. The other, a deletion variant in the exons 25–31, which was confirmed by site-specific analysis using quantitative polymerase chain reaction, was predicted to cause a significant in-frame deletion of amino acid residues in the encoded protein. No frequency was observed in the database of DECIPHER or ISCA. Segregation analysis revealed that the proband’s parents are heterozygous carriers for both variants. According to the ACMG guidelines, the nonsense variant was classified as pathogenic and the deletion variant was classified as likely pathogenic.

Based on the patient’s clinical manifestations, neurological examinations, blood smear, cranial MRI, and gene testing, he was diagnosed with ChAc. The patient was prescribed oral medication, including benzhexol 2 mg twice daily, clonazepam 1 mg twice daily, and aripiprazole 5 mg in the morning and 7.5 mg at night. Meanwhile, he received transcutaneous electrical nerve stimulation and interferential therapy. After 2 weeks of hospitalization, his perioral and appendicular chorea were significantly relieved. During a one-year follow-up via telephone, it was found that his symptoms had gradually improved, specifically with a decrease in the frequency and amplitude of involuntary limb movements compared to his initial presentation.

## Discussion and conclusion

ChAc is a rare hereditary autosomal recessive neurodegenerative disorder that is characterized by adult-onset chorea-like involuntary movement and caudate nucleus atrophy in the brain, which resembles that seen in Huntington’s disease. Distinguished from Huntington’s disease, ChAc presents with increased acanthocytes in peripheral blood. Patients may also show orofacial lingual dyskinesia, vocal tics, cognitive-psychiatric disorder, seizures, peripheral neuropathy, and increased serum biochemical indicators. There are no standard diagnostic criteria for ChAc, a combination of clinical features, laboratory findings, and neuroimaging can help support the diagnosis.

Detection of biallelic pathogenic *VPS13A* variants confirms the diagnosis of ChAc. The *VPS13A* gene encodes a protein called chorein, which is 250 kb in length and composed of 73 exons. Mutation of the *VPS13A* gene shows defects in intracellular transmembrane proteins transportation and vacuolar protein sorting [1, 4]. Immunoblot analysis of ChAc-expressed human tissues shows a high density of expected mutant sequence in the brain, heart, skeletal muscle, and kidney, giving a reasonable explanation for the myopathy and cardiomyopathy as well as neuropsychiatric symptoms of ChAc. Chorein also resides in the cell membrane of erythrocytes, interacting with β-adducin and β-actin to maintain the cytoskeleton [7]. Further, absent or reduced expression of the *VPS13A* causes a reduction in intracellular phosphatidylinositol-4-phosphate (PtdIns [4]) [8] levels, resulting in the destruction of the cell membrane and abnormal morphology of erythrocytes through unconjugated membrane components of the spectrin/actin cytoskeleton. Therefore, a Western blot screening test for the level of chorein will facilitate the diagnosis [9].

In this case, we reported a 29-year-old male patient with chronic onset and progressive involuntary movements of the lip, tongue, and limbs, accompanied by slurred speech, vocal tics, tongue and lip bites, and a shuffling gait. Blood routine showed increased RBC counts but small in cell size and low Hb content, which was suspected to be related to acanthocytosis. Acanthocytes are vulnerable to trap and destruction by the spleen due to their morphology [10]. Broken red cells showed in peripheral blood smear and elevated liver enzymes indicate a certain degree of liver insufficiency of the patient, which may be caused by chronic hemolysis [11]. The variants found in this patient have not been reported till now; however, previous studies on other variants may help us understand their potential pathogenicity [12–14]. It is likely that these variants cause premature translation termination, triggering the nonsense-mediated mRNA decay pathway and leading to degradation of the *VPS13A*-mRNA. Additionally, the variants may affect spliceosome formation, leading to abnormal mRNA splicing and production of abnormal chorein. These genetic changes are believed to contribute to the development of ChAc. The limitation of this study is that Western blot testing was not performed on the patient due to financial constraints. As a result, the impact of the identified variants on the expression and function of the chorein could not be directly assessed. [6].

In conclusion, this study reports two novel variants in *VPS13A* gene in a Chinese family: a heterozygous nonsense variant (c.8215G > T, p. Glu2739Ter) and a deletion variant in the exons 25–31. The identification of the newly discovered recessive variants in ChAc can provide valuable information for genetic counseling. Furthermore, this study sheds light on the pathogenic mechanisms underlying ChAc and can assist clinicians in accurate diagnosis and management.

## Data Availability

The clinical data underlying this case is collected from multiple hospitals. The datasets generated and analyzed during the current study are available from the corresponding author on reasonable request.

## List of abbreviations

ChAc: Chorea-acanthocytosis
VPS13A: Vacuolar Protein Sorting 13 homolog A, ABL:Abetalipoproteinemia
MLS: McLeod syndrome
HDL2: Huntington’s disease-like 2
PKAN: Pantothenate kinase-associated neurodegeneration
K-F: Kayser-Fleischer
MMSE: Mini-Mental State Examination
MRI: Magnetic resonance imaging
PtdIns[4]: Phosphatidylinositol-4-phosphate
ACMG: American College of Medical Genetics and Genomics
